# Construction and Immunogenicity of Novel Chimeric Virus-Like Particles Bearing Antigens of Infectious Bronchitis Virus and Newcastle Disease Virus

**DOI:** 10.3390/v11030254

**Published:** 2019-03-13

**Authors:** Xuan Wu, Xiwen Zhai, Yan Lai, Lei Zuo, Yu Zhang, Xueran Mei, Rong Xiang, Zhuangzhuang Kang, Long Zhou, Hongning Wang

**Affiliations:** School of Life Science, Sichuan University, Animal Disease Prevention and Food Safety Key Laboratory of Sichuan Province, “985 Project” Science Innovative Platform for Resource and Environment Protection of Southwestern, Key Laboratory of Bio-resources and Eco-environment, Ministry of Education, 29# Wangjiang Road, Chengdu 610065, China; wuxuan329@hotmail.com (X.W.); zhaixiwenOuO@163.com (X.Z.); llllaiyan@163.com (Y.L.); zuolei0104@foxmail.com (L.Z.); yuzhang712@163.com (Y.Z.); meixueran@163.com (X.M.); xxingxrong@163.com (R.X.); kang_zhuangzhuang@163.com (Z.K.); zhoulongscu@163.com (L.Z.)

**Keywords:** Infectious bronchitis virus, Newcastle disease virus, chimeric virus-like particles, bivalent vaccine

## Abstract

Infectious bronchitis virus (IBV) and Newcastle disease virus (NDV) are two poultry pathogens seriously affecting the poultry industry. Here, IBV S1 and the ectodomain of NDV F proteins were separately linked with the trans-membrane and carboxy-terminal domain of IBV S protein (S_TMCT_), composing rS and rF; thus, a novel chimeric infectious bronchitis-Newcastle disease (IB-ND) virus-like particles (VLPs) vaccine containing the rS, rF, and IBV M protein was constructed. Under the transmission electron microscope (TEM), VLPs possessing similar morphology to natural IBV were observed. To evaluate the immunogenicity of chimeric IB-ND VLPs, specific pathogen-free (SPF) chickens were immunized with three increasing doses (50, 75, and 100 μg protein of VLPs). Results of ELISAs detecting IBV and NDV specific antibodies and IL-4 and IFN-γ T cell cytokines indicated that vaccination with chimeric IB-ND VLPs could efficiently induce humoral and cellular immune responses. In the challenge study, chimeric IB-ND VLPs (100 μg protein) provided 100% protection against IBV or NDV virulent challenge from death, and viral RNA levels in tissues and swabs were greatly reduced. Collectively, chimeric IB-ND VLPs are highly immunogenic and could provide complete protection from an IBV or NDV virulent challenge. Chimeric IB-ND VLPs are an appealing vaccine candidate and a promising vaccine platform bearing multivalent antigens.

## 1. Introduction

Avian infectious bronchitis (IB) and Newcastle disease (ND) are both common, highly contagious, and acute avian diseases and have been causing heavy losses in the poultry industry [1,2]. Infectious bronchitis virus (IBV), the pathogen of IB, is a member of *Gammacoronavirus* of the *Coronaviridae* family (http://www.ictv.global). The genome of IBV is about 27.6 kb in length, encoding fifteen non-structural proteins and four structural proteins: Spike glycoprotein (S), small membrane protein (E), membrane glycoprotein (M), and phosphorylated nucleocapsid protein (N). The M glycoprotein, the most abundant protein in the viral envelope, performs core functions in the process of coronavirus assembly and budding [3,4]. Furthermore, the M protein is generally considered to be an essential component in the formation of coronavirus-like particles (CoVLPs) [5,6]. The S glycoprotein, which is post-translationally cleaved into two distinct functional subunits (S1 and S2), forms the distinctive spikes on the surface of IBV [7,8]. The S protein is responsible for receptor binding and determining host tropisms; in particular, the S1 subunit can elicit virus-neutralizing antibodies [1], while the S2 subunit anchors the S protein in the surface of the virion through the C-terminal trans-membrane domain (TM, aa1093-1136 of S protein of IBV) inlaid into the envelope and the carboxy-terminal domain (CT, aa1137-1162 of S protein of IBV) noncovalently interacting with the M glycoprotein [3,9]. ND is caused by virulent strains of Newcastle disease virus (NDV), which is a member of the genus *Avulavirus* under family *Paramyxoviridae* (http://www.ictv.global). NDV has a 15kb long genome comprising six genes which individually encode the nucleocapsid (N), matrix protein (M), phosphoprotein (P), fusion protein (F), haemagglutinin-neuraminidase protein (HN), and large polymerase protein (L) [10]. F and HN are two glycoproteins displayed on the virion surface. The F protein mediates virus fusion with the host cell membrane and plays the major role in the virulence of NDV strains. The HN protein assists the F protein in its function [2,11]. The F protein consists of an ectodomain (F_ecto_, aa1-499 of F protein of NDV) displayed on the viral envelope, a hydrophobic transmembrane domain (TM, aa500-523 of F protein of NDV), and a cytoplasmic domain (CT, aa524-553 of F protein of NDV) near the carboxyl terminus; similar to the IBV S protein, the TM/CT domain anchors the F protein in the surface of the virion [12]. F is thought to be the predominant antigen in NDV vaccine studies. Antibodies elicited by the F protein can protect chickens from lethal NDV challenge [2,13,14,15,16].

Nowadays, IBV and NDV are controlled using live-attenuated vaccines and inactivated vaccines [15,17]. Live-attenuated vaccines are thought to be the most effective vaccines. However, safety is the main concern about live vaccines. Live vaccines may cause diseases in immunocompromised individuals. Mutations in the genome of live vaccine strains could cause a reversion to virulence and further result in diseases in vaccinated individuals [18]. Novel IBV strains arising from genome recombination have been reported in recent years [19,20]. Genome recombination events between NDV vaccine strains and NDV wild strains were also observed [21,22]. In addition, NDV-attenuated vaccines may cause mild respiratory or gastrointestinal disease, resulting in weight loss, reduced egg production, and increased sensitivity to other pathogens [23]. Compared with live vaccines, inactivated vaccines are safer but induce weaker and shorter-lived immune responses; most notably, inactivated vaccines could not effectively stimulate cellular immune responses [17]. Furthermore, the potential for the incomplete inactivation of a virus can also result in disease in vaccinated individuals. According to these facts, developing novel effective vaccines against IBV and NDV is badly needed. Virus-like particles (VLPs) are empty shells composed of virus structural proteins (and a viral envelope in enveloped viruses), possessing similar morphology with the native viruses [24]. Due to the absence of virus genome, VLPs are noninfectious [24,25]. These qualities contribute to the effectiveness and safety of VLPs as vaccines [26,27]. In addition, antigens or epitopes from different pathogens can be simultaneously exhibited on the surface of VLPs through either recombinant DNA technology or chemical conjugation, making the chimeric VLPs a multi-antigenicity vaccine platform [24]. The first VLPs were constructed with the surface antigen of hepatitis B virus in 1982 [28]. To date, VLPs of various viruses had been constructed and applied as nonreplicating subunit vaccines against viral infection [25,26]. IBV VLPs have been shown to be a promising vaccine approach, and they possess the potential to carry other virus antigens to form multivalent vaccines [29,30].

In this study, F_ecto_ and the IBV S1 protein were fused to the TM and CT domain of the IBV S protein (S_TMCT_), forming the recombinant F (rF) and recombinant S (rS) protein, respectively, and chimeric infectious bronchitis-Newcastle disease (IB-ND) VLPs were constructed with these two recombinant proteins and IBV M proteins through the baculovirus system. Subsequently, the immunogenicity of the chimeric VLPs were evaluated as a vaccine in specific-pathogen-free (SPF) chickens.

## 2. Materials and Methods

### 2.1. Viruses and Cells

The IBV M41 and NDV La Sota and F48E9 strains were stored at −80 °C by Animal Disease Prevention and Food Safety Key Laboratory of Sichuan Province, Sichuan University, and propagated in 10-day-old embryonated SPF eggs (Boehringer Ingelheim Vital Biotechnology Co. Ltd., Beijing, China) when used as previously described [31]. Sf9 cells were cultured in Sf-900TM III SFM (Gibco, Grand Island, NY, USA) at 27 °C.

### 2.2. Construction of rS and rF Genes

**Figure 1:**
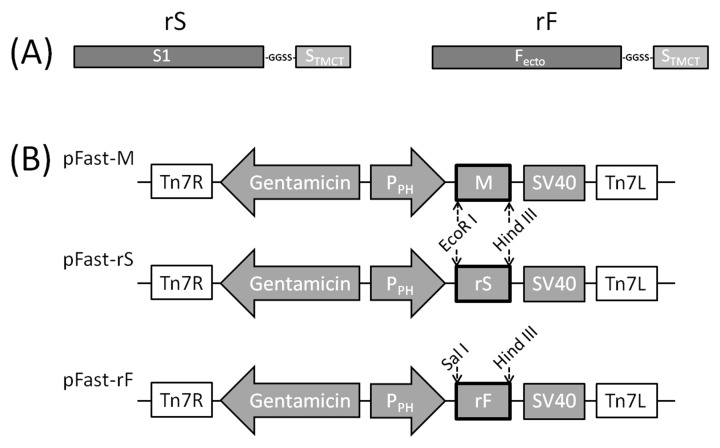
rS and rF genes and recombinant plasmids. (**A**) Schematic diagram for the recombinant S and recombinant F genes; “-GGSS-” indicates the flexible peptide (GlyGlySerSer). (**B**) The P_ph_ is polyhedrin promoter, and M, rS, and rF genes are individually under the control of P_ph_, restriction enzyme sites, which are marked with dotted lines. SV40, SV40: Polyadenylation signal. Tn7R and Tn7L: Right and left elements of the Tn7 transposon.

The RNA of the viruses was extracted with TRIzol (Invitrogen, Carlsbad, CA, USA). Subsequently, cDNA was synthetized using the SuperScript III FirstStrand Synthesis System (TaKaRa, Kyoto, Japan) following the manufacturer’s instructions. Briefly, the reverse transcription reaction mixture consists of 2 μL 5 × PrimeScript Buffer, 0.5 μL PrimeScript RT Enzyme Mix I, 0.5 μL Oligo dT Primer, 0.5 μL Random 6 mers, the RNA of the virus, and RNase Free dH2O, thus creating a final volume of 10 μL. S1 and S_TMCT_ were amplified with IBV M41 cDNA, and F_ecto_ was amplified with NDV La Sota cDNA. S1 and F_ecto_ were individually linked with S_TMCT_ by digestion-ligation, thus forming the rS and rF genes (Figure 1A).

To make these three domains function naturally, a sequence encoding a short flexible peptide (GlyGlySerSer) was inserted at the fusion site. The sequences of primers used in this step are listed in Table 1.

### 2.3. Construction of Recombinant Baculoviruses Expressing rS, rF and M Genes

rS, rF, and M genes were individually cloned into the *pEASY*^®^-T1 vector (Transgen, Beijing, China). Subsequently, rS and M were individually subcloned into the pFastBac1 plasmid (Invitrogen, Carlsbad, CA, USA) at the *Eco*R I/*Hin*d III site, and rF was subcloned into pFastBac1 at the *Sal* I/*Hin*d III site (Figure 1B). The recombinant pFastBac1 vectors were chemically transformed into DH10Bac^TM^ *E. coli* (Invitrogen, Carlsbad, CA, USA) to generate recombinant bacmids (rBMs). Positive clones were verified by blue/white selection and sequencing using M13 primers. rBM-rF, rBM-rS, and rBM-M were purified with the PureLink^TM^ HiPure Plasmid DNA Miniprep Kit (Invitrogen, Carlsbad, CA, USA) according to the manufacturer’s instructions, after which 2 μg of purified rBMs and 8 μL of Cellfectin^®^ II Reagent were individually diluted in 100 μL Grace’s Medium. Afterwards, the diluted DNA and diluted Cellfectin^®^ II were combined to make the rBMs packaged in lipidosome. After incubating for 10 min, the transfection mixture was added dropwise onto sf9 cells cultured in 6-well plate at a density of 8 × 10^5^ per well. After incubating at 27 °C for 5 h, the transfection mixture was replaced by 2 mL of Sf-900TM II SMF (Gibco, Grand Island, NY, USA). Signs of viral infection were observed 72 h post transfection. Cells were frozen-and-thawed twice to release the recombinant baculoviruses (rBVs). The culture medium was collected and centrifuged at 500 × g for 5 min to remove cell debris; thus, the first passages of rBV-rF, rBV-rS, and rBV-M were obtained. The titers of rBVs were determined by plaque assays according to the manual of baculovirus system (Invitrogen, Carlsbad, CA, USA).

### 2.4. Western Blot Analysis of Protein Expression

To express target proteins, sf9 cells were separately infected by the third passage of rBV-rF, rBV-rS, and rBV-M (with titers of 3 × 10^7^–6 × 10^7^ pfu/mL) at a multiplicity of infection (MOI) of 5. Seventy-two hours post-infection, cell pellets were lysed by RIPA (Beyotime, Shanghai, China), and the cytolysate—Together with supernatant culture media—Were analyzed by the western blot. Rabbit polyclonal sera against IBV was used as the primary antibody to detect rS and M, and rabbit anti-Newcastle disease polyclonal antibody (Absin Bioscience, Shanghai, China) was used to detect rF. Anti-rabbit IgG HRP-linked antibody was used as the secondary antibody. The bands were visualized using the DAB Substrate kit (Solarbio, Beijing, China) following the manufacturer’s instructions.

### 2.5. Production and Purification of VLPs

For VLPs production, sf9 cells were co-infected with rBV-rF, rBV-rS, and rBV-M at a MOI of 5. After 96 h, the culture medium was collected and centrifuged at 4000 × *g* for 20 min at 4 °C to remove cell debris; subsequently, the supernatants were ultracentrifuged at 80,000 × g for 1.5 h at 4 °C. After resuspending sediments in PBS, the solution was loaded on a 20%–30%–40%–50% (*w*/*v*) discontinuous sucrose gradient and ultracentrifuged at 80,000 × *g* for 5 h at 4 °C. Purified VLPs at the interface between 30% and 40% were collected. After purification, 297–423 μg of VLPs could be harvested from the 100 mL culture medium. SDS-PAGE was performed to analyze the VLPs samples. Proteins were stained with Coomassie brilliant blue (CBB). The total protein concentrations of VLPs, inactivated IBV, and inactivated NDV were determined by the Bradford Protein Assay Kit (Beyotime, Shanghai, China). The concentrations of rS and rF proteins in VLPs, S1 protein in inactivated IBV, and F protein in inactivated NDV were determined by the densitometry of CBB-stained SDS-PAGE gel with BSA as standard.

To observe the chimeric IB-ND VLPs, purified VLPs were loaded onto a carbon-coated copper grid for 5 min, negatively-stained with 2% (*w*/*v*) phosphotungstic acid for 1 min, and then viewed under TEM (Tecnai G2 F20 S-TWIN, Hillsboro, OR, USA).

### 2.6. Immunization and Challenge

In the first animal study, 100 7-day-old SPF chickens (Boehringer Ingelheim Vital Biotechnology Co. Ltd., Beijing, China) were randomly divided into 10 groups, 10 chickens per group. Chickens in group 1 and 2 were immunized with VLPs containing 50 μg total of proteins (7.3 μg rS and 6.6 μg rF) per chicken. Chickens in group 3 and 4 were immunized with VLPs containing 75 μg total of proteins (10.9 μg rS and 10.0 μg rF) per chicken. Chickens in group 5 and 6 were immunized with VLPs containing 100 μg total of proteins (14.5 μg of rS and 13.3 μg of rF) per chicken. Chickens in group 7 and 8 were inoculated with inactivated IBV M41 (14.4 μg of S1 protein, 10^7.3^ EID_50_) and inactivated NDV La Sota (13.1 μg of F protein, 10^8.9^ EID_50_) with ISA206 adjuvant via the intramuscular route, respectively. M41 and La Sota strains were inactivated by formaldehyde with a final concentration of 0.2% at 37 °C for 24 h. Chickens in group 9 and 10 were mock control injected with culture medium from sf9 cells infected with wild-type baculovirus, and these two groups were set as the IBV or NDV infection control in the challenge study. On the 14th day-post-primary-vaccination (dpv), boost vaccinations were performed with the same program and doses as the primary vaccination. On the 28th dpv, chickens in odd groups were intranasally challenged with 10^6.7^ EID_50_ IBV M41, and chickens in even groups were intranasally challenged with 10^6.4^ EID_50_ NDV F48E9.

In the second animal study to measure viral RNA levels in tissues, 40 7-day-old SPF chickens were randomly divided into 10 groups (*n* = 4). The immune and challenge programs were the same as that in the first study.

### 2.7. Sample Collection

In the first animal study, chickens were bled to measure IBV and NDV specific antibody titers on the 7th, 14th, 21st, and 28th dpv. Serum samples on the 14th and 28th dpv were also used to detect the concentrations of IL-4 and IFN-γ. Oral swabs were collected from the surviving animals on the 2nd, 4th, 6th, 8th, and 10th dpv to monitor virus shedding. After being challenged, chickens were observed daily for 15 days to check clinical symptoms.

In the second animal study, on the 5th day-post-challenge (dpc), surviving chickens were sacrificed to get tissue samples for the quantification of the replication of challenged viruses. Lungs, tracheas, spleens, kidneys, and small intestines were taken to determine IBV RNA levels; lungs, tracheas, spleens, brains, and small intestines were taken for NDV RNA level determinations.

The animal experiments in this study were approved by the Animal Ethics Committee (ACE) of the College of Life Sciences, Sichuan University (license: SYXK-Chuan-2013-185). All experiment procedures and animal welfare standards strictly followed the guidelines of Animal Management at Sichuan University.

### 2.8. Evaluation of Vaccine Efficacy

IBV-specific antibodies in serum were detected by the Infectious Bronchitis Virus Antibody Test Kit (IDEXX, Westbrook, ME, USA); NDV antibodies were detected by the Newcastle Disease Virus Antibody Test Kit (IDEXX, Westbrook, ME, USA). The concentrations of IL-4 and IFN-γ in serum were individually monitored using an ELISA Kit for Interleukin 4 (Cloud-Clone, Houston, TX, USA) and an ELISA Kit for Interferon Gamma (Cloud-Clone, Houston, TX, USA) according to the manufacturer’s instructions.

To evaluate the viral RNA levels in tissues, 0.1 g of each tissue sample was used for RNA isolation. The swabs were suspended in 1 mL PBS; subsequently, 200 μL of supernatant from each sample was used to extract RNA for the evaluation of viral RNA levels in swabs. After reverse transcription (total volume of 10 μL), 1 μL of reverse transcription products (i.e., one-tenth of the total RNA) was used as template in absolute quantification real-time PCRs (qPCR) to determine IBV and NDV copy numbers using the SsoFast^TM^ EvaGreen^®^ Supermix (BIO-RAD, Berkeley, CA, USA). Primers were designed via Primer Premier version 6 (Premier Inc., Palo Alto, CA, USA) according to the genome sequences of IBV M41 (GenBank accession No. AY851295.1) and NDV F48E9 (GenBank accession No. MG456905). Primer sequences were as follows: IB-sense, 5′-TCCAGAACCACCACCATT-3′; IB-antisense, 5′-TGTCACACTCCTCAGCAT-3′; ND-sense, 5′-CAGCGTCTTGACTTGTGGACAGAT-3′; and ND-antisense, 5′-ATGCCGACAGCGACTTCTTCATC-3′. All the qPCRs were carried out quadruplicately.

### 2.9. Statistical Analysis

Statistical significance differences in serological and viral RNA level analyses between groups were evaluated by Student’s *t*-test with GraphPad Prism version 6 (GraphPad Software Inc., La Jolla, CA, USA). Differences were considered to be significant at * *p* < 0.05 or ** *p* < 0.01.

## 3. Results

### 3.1. Western Blot Analyses of rS, rF, and M Protein Expression

**Figure 2:**
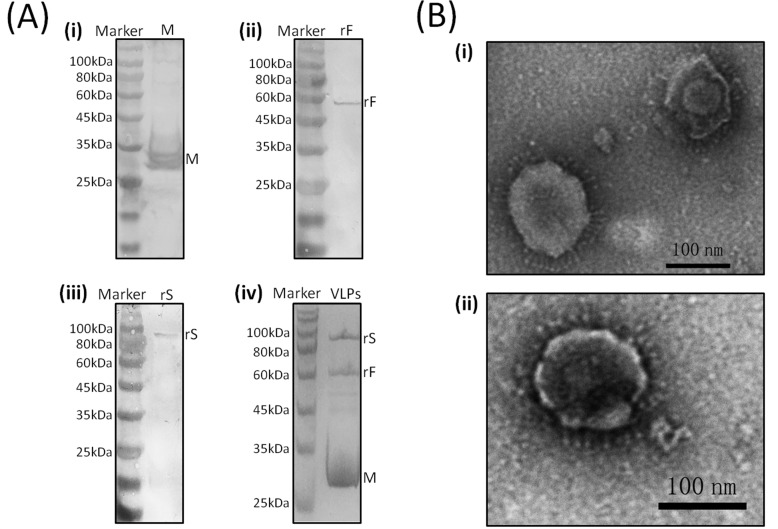
Expression of M, rS, and rF proteins and characterization of chimeric infectious bronchitis-Newcastle disease (IB-ND) virus-like particles (VLPs). (**i**–**iii**) in (**A**) are individual western-blot results of M, rF, and rS proteins using anti-infectious bronchitis virus (IBV) or Newcastle disease virus (NDV) serum; (**iv**) is the SDS-PAGE result of VLPs. Protein molecular weight standards (Marker) and positions of M, rS, and rF are indicated. (**B**) Phosphotungstic acid negative stain of TEM images of (**i**) chimeric IB-ND VLPs and (**ii**) native IBV particles. The scale is marked on the lower right corner.

An about 90 kDa band and bands between 25–35 kDa were separately detected by anti-IBV sera in rBV-rS and rBV-M infected samples; an approximately 60 kDa band was detected by an anti-NDV antibody in the rBV-rF infected sample. The results showed that rS, rF, and M proteins could be expressed under current conditions (Figure 2A). In addition, the predicted sizes of rS, rF, and M proteins are 66.5 kDa, 60.7 kDa, and 25.6 kDa, respectively. According to the results of the western blot, the rS and M proteins expressed in this study were larger than predicted, and the rF protein was similar to predicted seize, indicating that rS and M might be glycosylated while rF might not be glycosylated.

### 3.2. Characterization of Chimeric IB-ND VLPs

Purified VLPs were analyzed by SDS-PAGE, and three distinct protein bands were observed: An about 90 kDa band corresponded to rS, a 60 kDa band corresponded to rF, and a 25–35 kDa band corresponded to M (Figure 2A).

Under TEM, the morphology of chimeric IB-ND VLPs was observed, as shown in Figure 2B; the chimeric VLPs appeared to be spherical particles with a diameter of about 100 nm, which were similar to native IBV.

### 3.3. Evaluation of IBV- and NDV-Specific Antibodies

**Figure 3:**
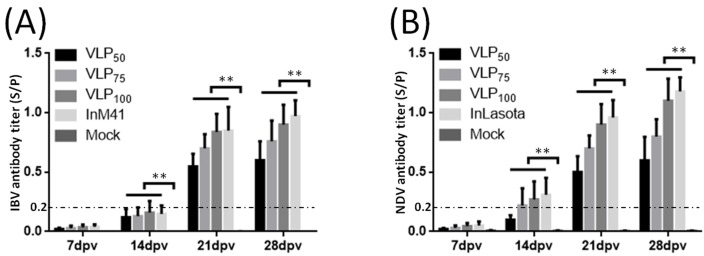
IBV- and NDV-specific antibody titers in serum. (**A**,**B**) are separate antibody titers against IBV and NDV. Titers are shown as mean S/P values + S.D. of each group. The threshold cut-off values of IBV and NDV ELISAs are both 0.2. S/P = [Sample A(650)–NC A(650)]/[PC A(650)—NC A(650)]. * and ** indicate *p* < 0.05 and *p* < 0.01, respectively.

On the 7th, 14th, 21st, and 28th dpv, levels of IBV- and NDV-specific antibodies in serum were evaluated by indirect ELISAs. On the 14th dpv, very significantly higher mean IBV- and NDV-specific antibody titers in serum from immunized chickens than that from mock chickens were observed (*p* < 0.01) (Figure 3). The mean antibody titers apparently increased following boost vaccination. Antibody levels induced by VLPs showed dose-dependent increases within current immunization dose range. As for comparison between VLPs and inactivated vaccine groups containing similar doses of antigen proteins (VLPs_100_ versus InM41/InLa Sota), no significant differences were observed.

### 3.4. Cytokines Induction in Serum of Immunized Chickens

**Figure 4:**
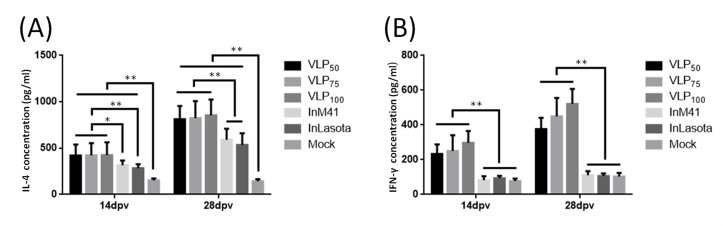
(**A**) IL-4 and (**B**) IFN-γ concentrations in serum on the 14th and 28th day-post-primary-vaccination (dpv). Concentrations are shown as mean values + S.D. of each group. * and ** indicate *p* < 0.05 and *p* < 0.01, respectively.

The ability of chimeric IB-ND VLPs in inducing cellular immune response was investigated by measuring the concentrations of IL-4 and IFN-γ in serum from immunized chickens on the 14th and 28th dpv. As Figure 4A shows, the mean concentrations of IL-4 in serum of chickens from all vaccinated groups were very significantly higher compared with that from Mock group (*p* < 0.01). Furthermore, VLPs induced higher IL-4 levels than inactivated vaccines did, especially on the 28th dpv. IL-4 levels in serum of chickens from VLP groups were very significantly higher than that from the two inactivated vaccine groups (*p* < 0.01) (Figure 4A).

As for IFN-γ, no significant levels of IFN-γ were detected in serum from inactivated vaccines-injected chickens compared with that in serum of chickens from Mock group, indicating that the two inactivated vaccines failed to induce IFN-γ production (Figure 4B). In contrast, VLPs stimulated very significantly higher IFN-γ levels than the inactivated vaccines did (*p* < 0.01).

### 3.5. Evaluation of Protection Against IBV or NDV Challenge

**Figure 5:**
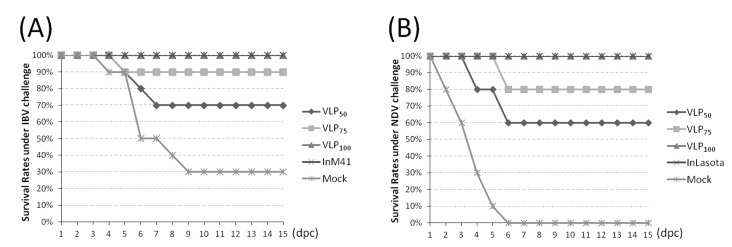
Survival rates of chickens in different groups against virulent challenge. Chickens were monitored for survival after the (**A**) IBV and (**B**) NDV virulent challenge for 15 days.

In the first animal study, chickens were challenged with virulent IBV or NDV strains and kept for 15 days to observe clinical symptoms and monitor the virus shedding in oral swabs. Under the virulent IBV strain challenge, 3 chickens showed apparent symptoms in the VLP_50_ group including depression and huddling, and these 3 chickens died on the 5th, 6th, and 7th dpc, respectively. In the VLP_75_ group, only 1 chicken showed symptoms and died on the 5th dpc; chickens in the VLP_100_ and InM41 groups showed complete resistance to virulent IBV challenge, and no apparent symptoms were observed. In contrast, all chickens in the Mock group showed symptoms, and 8 of them showed severe depression. Finally, only 3 chickens survived (Figure 5A). Under the NDV challenge, 6 chickens in the VLP_50_ group showed symptoms, and 4 of them showed neurological signs. These 4 chickens died within 6 days; in the VLP_75_ group, 2 chickens showed neurological signs and died on the 6th dpc. No chickens in the VLP_100_ group showed symptoms, and all chickens survived. 2 chickens in the InLa Sota group were observed to have mild depression, and no chickens died. All chickens in the Mock group showed severe depression and neurological signs and died within 6 days (Figure 5B).

**Figure 6:**
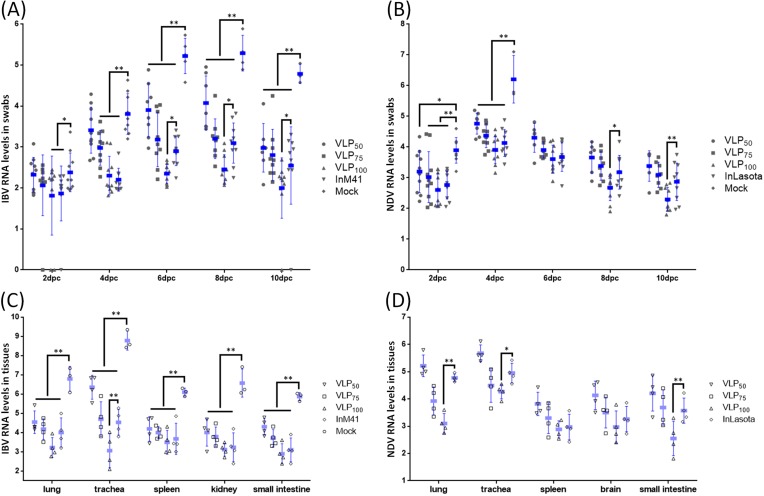
IBV and NDV RNA levels in swabs and tissues. (**A**,**B**) are, separately, the mean IBV and NDV RNA levels per reaction in swabs. (**C**,**D**) are, individually, the IBV and NDV RNA levels per reaction in tissues from the surviving animals. Data are plotted for individual chickens and overlaid with the mean titers ± S.D. * *p* < 0.05, ** *p* < 0.01. All data are summarized as the log of the viral RNA copy numbers.

Oral swabs were collected on the 2nd, 4th, 6th, 8th, and 10th dpc for viral RNA level monitoring. As Figure 6A shows, the mean IBV RNA levels in the swabs of chickens from immunized groups were reduced with different degrees compared with that from the Mock group at all five time points. On the 2nd dpc, the mean IBV RNA levels in swabs of chickens from VLP_100_ and InM41 groups were significantly lower than those from the Mock group (*p* < 0.05); on the 4th dpc, the mean IBV RNA levels in swabs from VLP_75_, VLP_100_, and InM41 were very significantly reduced compared with that from Mock group (*p* < 0.01). At the last three time points, the mean IBV RNA levels in swabs from all vaccinated groups were very significantly lower than that from the Mock group (*p* < 0.01). Furthermore, the mean IBV RNA levels in swabs from the VLP_100_ group were significantly lower than that of the InM41 group on the 6th, 8th, and 10th dpc (*p* < 0.01). As for NDV, the mean RNA level in swabs from the VLP_50_ group was significantly lower than that from the Mock group on the 2nd dpc. The mean NDV RNA levels in swabs from all vaccinated groups were very significantly lower than that from the Mock group on the 4th dpc; at the last three time points, no difference analyses between swabs from immunized chickens and Mock chickens were conducted, because all chickens died in the Mock group (Figure 6B). Furthermore, on the 8th dpc, the mean NDV RNA level in swabs from the VLP_100_ group was significantly lower than that from the InLa Sota group (*p* < 0.05), and on the 10th dpc, the difference between the mean NDV RNA levels in swabs from VLP_100_ and InLa Sota was very significant (*p* < 0.01).

In the second animal study, IBV and NDV RNA levels in tissues were measured on the 5th dpc. In general, the mean IBV RNA levels in tissues of chickens from immunized groups were very significantly reduced compared with the Mock group (*p* < 0.01). Furthermore, the mean IBV RNA level in tracheas of chickens from VLP_100_ was very significantly lower than that from InM41 (*p* < 0.01) (Figure 6C). In the NDV challenge study, all 4 chickens in the Mock group died before the 5th dpc. Difference analyses were conducted between the VLP_100_ and InLa Sota groups. The mean NDV RNA levels in the lungs and small intestines of chickens from VLP_100_ were very significantly lower than that from InLa Sota (*p* < 0.01), and the mean NDV RNA level in tracheas of chickens from the VLP_100_ group was significantly lower than that from the InLa Sota group (Figure 6D).

## 4. Discussion

IBV S1 and NDV F proteins are both promising candidate antigens for the development of novel recombinant vaccines, and it has been proved that these two proteins are able to provide efficient protection against the IBV or NDV virulent challenge [1,15,32,33]. Vaccination with multivalent vaccines has potential advantages in reducing costs and labor. Conventional live and inactivated vaccines against IBV and NDV are widely used in the field. Nevertheless, both live and inactivated vaccines have some obvious shortcomings. Mutants in the genome of viruses in live vaccines—as well as recombination between vaccine viruses and wild viruses—could cause the emergence of novel strains and further result in diseases in vaccinated individuals [18]. In addition, NDV live vaccines sometimes cause mild clinical symptoms [23]. Inactivated vaccines are thought to be safer than live vaccines; however, the potential for incomplete inactivation of the virus could also cause diseases in vaccinated individuals [18]. Besides, inactivated vaccines are not effective in stimulating cellular responses. To better control IBV and NDV, several novel vaccines against these viruses have been developed, among which reverse genetic vaccines are high-profile [32,34]. Particularly, NDV reverse genetic vaccines (live or inactivated) have been licensed for use in the field. However, safety is still the major concern of reverse genetic vaccines. As mentioned above, when these reverse genetic strains are used as live vaccines, mutants—as well as recombination—are likely to result in the emergence of novel strains. In contrast, lacking viral RNA makes the VLPs non-infectious and eliminates the possibilities of mutants and recombination with wild strains, which could happen when live vaccines are used. Moreover, compared with inactivated vaccines, VLPs are more similar to infectious viruses in terms of structure and morpholog, so VLPs could induce stronger immune responses than inactivated vaccines do [25]. VLPs are considered the most suitable multivalent vaccine platform for RNA viruses with high mutation rates [24,25]. The M protein has been proved to be essential in the construction of CoVLPs [5,29]. In order to make the NDV F protein interact with the IBV M protein and further take part in the construction of VLPs, the TMCT domain of the F protein was replaced by that of the IBV S protein, thus forming the rF protein. Afterwards, the co-expression of the IBV M and rS proteins and the NDV rF protein in insect cells resulted in the generation of spherical particles possessing similar size and shape to native IBV. SDS-PAGE analysis of the purified spherical particles showed that they were composed of M, rS, and rF proteins, indicating that chimeric IB-ND VLPs bearing IBV and NDV antigens were successfully produced (Figure 2A).

To investigate whether chimeric IB-ND VLPs were efficient in inducing immune responses, purified VLPs were injected into chickens. Antibodies and cytokines in serum were measured. As for inducing humoral immune response, the chimeric IB-ND VLPs could effectively induce both IBV- and NDV-specific antibodies, and, even without adjuvant, VLP_100_ induced comparable antibody levels as inactivated vaccines did (with adjuvant). In nonreplicating vaccines, adjuvants serve as critical components to elicit adequate immune response [35]; VLPs, possessing similar morphology as the native viruses did, can be efficiently taken up by antigen presenting cells and processed in the same way as the native viruses are without any adjuvants [27]. As for cellular immune responses, the induction of significantly higher levels of IL-4 and IFN-γ than the Mock group demonstrates the ability of chimeric IB-ND VLPs to evoke both Th1- and Th2-type cellular immune responses (Figure 4). In contrast, the inactivated vaccines only induced significant IL-4 level compared with the Mock group; that is, only the Th2-type cellular immune response was induced [29]. Due to their structural and morphological similarity to infectious viruses and their ability to bind and penetrate host cells, VLPs could induce stronger cellular immune responses than inactivated vaccines did [25].

A quality vaccine should be able to prevent pathogen transmission. Our data showed that IBV shedding was efficiently suppressed in all vaccinated groups compared with the Mock group at all five time points, and NDV shedding was also efficiently suppressed on the 2nd and 4th dpc (Figure 6). In addition, significantly lower mean IBV RNA levels in swabs from VLP_100_ compared with that from the InM41 group were observed on the 6th, 8th, and 10th dpc (*p* < 0.01) (Figure 6A); the mean NDV RNA level differences between VLP_100_ and InLa Sota were individually significant (*p* < 0.05) and very significant (*p* < 0.01) on the 8th and 10th dpc. In tissues of challenged chickens, the mean IBV RNA level in tracheas and mean NDV titers in lungs, tracheas, and small intestines of chickens immunized with 100 μg of VLPs were lower than that of chickens immunized with inactivated vaccines (significant or very significant differences were observed). These results indicated that VLPs performed better than inactivated vaccines did. It is generally considered that the stronger humoral and cellular immunity resulted in less virus replication [36]. The antibody levels in serum of chickens from VLP_100_ were comparable to that from inactivated vaccine groups, so the lower virus titers in VLP_100_ groups are due to the stronger cellular immune responses induced by VLPs. In line with the results of the detection of cytokines in serum, VLPs were able to evoke both Th1- and Th2-type responses, while the inactivated vaccines only evoke Th2-type responses. Th1/Th2 mixed response is generally considered to be more preferable than a T2-type for preventing and treating viral infection [37,38]. In addition, the multivalent display and highly ordered structure present on surface of VLPs were thought to be a kind of pathogen associated molecular patterns (PAMPs) which can be recognized by Toll-like receptors (TLRs) and other pattern-recognition receptors (PRRs) [39], resulting in increased immunogenicity [25]. Due to the high levels of humoral and cellular responses induced by VLPs, after 15-day observation, no chickens immunized with 100 μg of VLPs died, and no symptoms were observed under the IBV or NDV virulent challenge, thus indicating that chimeric IB-ND VLPs are able to provide complete protection for chickens. Taken together, these data indicate that chimeric IB-ND VLPs are able to simultaneously induce IBV- and NDV-antibodies in levels which are comparable to that induced by single IBV or NDV inactivated vaccine (with adjuvant), and chimeric IB-ND VLPs performed better in inducing cellular responses and suppressing virus replication than inactivated vaccines did.

In this study, chimeric IB-ND VLPs were constructed using the IBV M protein and two recombinant proteins, i.e., rS and rF. When used as a vaccine, chimeric IB-ND VLPs were able to efficiently induce humoral and cellular immunity responses, and, when compared with inactivated vaccines, chimeric IB-ND VLPs performed better in terms of inducing cellular response and suppressing virus replication. In conclusion, this study indicated that IB VLPs can be used as a molecular platform for the genetic fusion of heterologous antigens. This study also suggested the promise of chimeric IB-ND VLPs as an appealing vaccine candidate which could simultaneously prevent IBV and NDV.

## Figures and Tables

**Table 1:** Primer sequences.

Primer	Sequence (5′→3′)	Genomic Position of Primers (nt)	Length of Product (bp)
S1-F	GAATTCATGTTGGTAACACCTCTTTTACTAG	20374—20398	1620
S1-R	CGCGGATCC*TCC*ACGACGTGTTCCATTA	21975—21960	
F_ecto_-F	GTCGACATGGGCTCCAGACCTTCTACC	4544—4564	1515
F_ecto_-R	CGCGGATCC*TCC*AGATGTGCTAGTCAG	6040—6026	
S_TMCT_-F	CGCGGATCC*TCA*TGGTATGTGTGG	23653—23664	228
S_TMCT_-R	AAGCTTTTAAACAGACTTTTTAGGTCTG	23862—23841	
M-F	GAATTCATGTCCAACGAAACAAATTGTAC	24511—24533	690
M-R	AAGCTTTTATGTGTAAAGGCTACTTCCACCTG	25188—25163	

Underlined sequences are cleavage sites of restriction enzyme. “CGC” in boxes are protection bases. Italic “*TCC*” and “*TCA*” are inserted codons. “*TCC*” is the reverse complement to the glycine codon “*GGA*” glycine (Gly) and “*TCA*” encodes serine (Ser), and these codons, together with “GGATCC” (*Bam*H I site), encode flexible peptide “GlyGlySerSer“. Genomic positions of primers are listed according to complete genome sequences of IBV M41 (AY851295.1) and NDV La Sota (AF077761.1).
